# Simple Synthesis of Red Iridium(III) Complexes with Sulfur-Contained Four-Membered Ancillary Ligands for OLEDs

**DOI:** 10.3390/molecules26092599

**Published:** 2021-04-29

**Authors:** Meng-Xi Mao, Fang-Ling Li, Yan Shen, Qi-Ming Liu, Shuai Xing, Xu-Feng Luo, Zhen-Long Tu, Xue-Jun Wu, You-Xuan Zheng

**Affiliations:** 1 State Key Laboratory of Coordination Chemistry, Collaborative Innovation Center of Advanced Microstructures, Jiangsu Key Laboratory of Advanced Organic Materials, School of Chemistry and Chemical Engineering, Nanjing University, Nanjing 210023, China; mf1924021@smail.nju.edu.cn (M.-X.M.); mg1824034@smail.nju.edu.cn (F.-L.L.); mf1824030@smail.nju.edu.cn (Y.S.); 171850043@smail.nju.edu.cn (Q.-M.L.); mf20240035@smail.nju.edu.cn (S.X.); dg1924062@smail.nju.edu.cn (X.-F.L.); dg1824074@smail.nju.edu.cn (Z.-L.T.); xjwu@nju.edu.cn (X.-J.W.); 2 Green Catalysis Center and College of Chemistry, Zhengzhou University, Zhengzhou 450001, China

**Keywords:** red iridium(III) complex, Ir-S-C-S four-membered backbone, fast synthesis, OLED

## Abstract

Phosphorescent iridium(III) complexes have been widely researched for the fabrication of efficient organic light-emitting diodes (OLEDs). In this work, three red Ir(III) complexes named Ir-1, Ir-2, and Ir-3, with Ir-S-C-S four-membered framework rings, were synthesized efficiently at room temperature within 5 min using sulfur-containing ancillary ligands with electron-donating groups of 9,10-dihydro-9,9-dimethylacridine, phenoxazine, and phenothiazine, respectively. Due to the same main ligand of 4-(4-(trifluoromethyl)phenyl)quinazoline, all Ir(III) complexes showed similar photoluminescence emissions at 622, 619, and 622 nm with phosphorescence quantum yields of 35.4%, 50.4%, and 52.8%, respectively. OLEDs employing these complexes as emitters with the structure of ITO (indium tin oxide)/HAT-CN (dipyra-zino[2,3-f,2′,3′-h]quinoxaline-2,3,6,7,10,11-hexacarbonitrile, 5 nm)/TAPC (4,4′-cyclohexylidenebis[*N*,*N*-bis-(4-methylphenyl)aniline], 40 nm)/TCTA (4,4″,4″-tris(carbazol-9-yl)triphenylamine, 10 nm)/Ir(III) complex (10 wt%): 2,6DCzPPy (2,6-bis-(3-(carbazol-9-yl)phenyl)pyridine, 10 nm)/TmPyPB (1,3,5-tri(mpyrid-3-yl-phenyl)benzene, 50 nm)/LiF (1 nm)/Al (100 nm) achieved good performance. In particular, the device based on complex Ir-3 with the phenothiazine unit showed the best performance with a maximum brightness of 22,480 cd m^−2^, a maximum current efficiency of 23.71 cd A^−1^, and a maximum external quantum efficiency of 18.1%. The research results suggest the Ir(III) complexes with a four-membered ring Ir-S-C-S backbone provide ideas for the rapid preparation of Ir(III) complexes for OLEDs.

## 1. Introduction

Since the innovative work in 1997 by C. W. Tang, organic light-emitting diodes (OLEDs) have been rapidly developed for decades [1]. Among them, phosphorescent OLEDs incorporating phosphorescent transition metal complexes such as iridium(III) or platinum(II) have attracted considerable attention from both academic and industrial communities, which is due to their superior efficiency, low turn-on voltage, and vibrant color tunability [2]. Introducing the central heavy atom in luminescent complexes can result in strong spin-orbit coupling (SOC), which would promote a triplet to ground radiative transition, harvesting both singlet/triplet excitons and leading to a quite high phosphorescence quantum efficiency [3,4]. Thus, a great deal of effort has been spent inquiring into the third-row transition-metal complexes to develop phosphorescent OLEDs with improving device performances [5]. In particular, the Ir(III) complexes with diverse ligands are by far the most effective heavy metal complexes of triplet emitters for phosphorescent OLEDs due to the relative short excited lifetime and high efficiency [6,7].

However, in most cases, the ancillary ligands of Ir(III) complexes mainly rely on six- or five-membered ring structures such as acetylacetonate (acac), picolinate (pic), and tetraphenylimidodiphosphinate [8]. Four-membered ancillary ligands are rarely researched because the conventional idea believes that the materials containing four-membered rings are not stable. The four-membered metal-ligand frame can shorten the distance between the metal center and ligand, so the MLCT (metal-to-ligand charge transfer) progress can be promoted. Therefore, it is a hopeful way to synthesize superior Ir(III) complexes by using four-membered ring ligands, which are beneficial to enhancing the photoluminescence (PL) quantum efficiency [9].

As a d^6^ metal, an iridium atom can form an octahedral coordination configuration in a trivalent form, and the key ligands in the Ir(III) complex generally coordinate to the metal center via the formation of Ir-N and Ir-C bonds [6,10]. The S atom has a high affinity and good coordination ability for transition metal ions, which can lead to the synthesis of Ir(III) complexes with sulfur-containing ligands at room temperature [11,12]. Besides, the sulfur atom is able to stabilize metal ions in unusual oxidation states [13,14,15]. Therefore, it has a great advantage over the costly traditional way of refluxing the [(C^N)_2_Ir(μ-Cl)]_2_ chloride-bridged dimers with cyclometalated or ancillary ligands at high temperature for a long time [16].

There are only a few examples of Ir(III) complexes with Ir-S-C-S structures and most of them are based on *N*,*N*′-diehyldithiocarbamate (Et_2_dtc) ancillary ligands. In 2005, Yang et al. first reported an Ir(III) (ppy)_2_Ir(Et_2_dtc) complex with a four-membered Ir-S-C-S structure [17]. Immediately afterwards, they reported more four-membered Ir(III) complexes, and the device based on a blue Ir(III) complex with a 1,3,4-oxadiazole derivative as cyclometalated ligand showed a maximum current efficiency (*η*_c,max_) of 9.88 cd A^−1^ [18,19]. In 2016, Mei et al. reported an orange-emitting complex Ir(dpp)_2_(dta) with diethyldithiocarbamate (dta) as the ancillary ligand, and its device achieved a maximum external quantum efficiency (EQE_max_) of 9.28% [20]. However, they prepared the Ir(III) complexes with the traditional method and the device performances should be improved. In our former study, we prepared some red Ir(III) complexes using 4-(4-(trifluoromethyl)phenyl)quinazoline (4tfmpq) as the cyclometalated ligand. Diisopropylamine, diphenylamine, carbazole and their derivatives substituted dithiocarbamates as ancillary ligands at room temperature within 10 min, and the device performances were improved greatly [16,21,22]. Furthermore, from our investigation we found that the Ir-S-C-S structures were only valuable for the red Ir(III) complexes with excellent device performance.

With the above consideration, three four-membered red Ir(III) complexes with sulfur atom-containing dithiocarbamate derivatives as ancillary ligands constructed with different electron-donating groups of 9,10-dihydro-9,9-dimethylacridine, phenoxazine, and phenothiazine were synthesized with a [(4tfmpq)_2_Ir(μ-Cl)]_2_ dimer at room temperature within 5 min. The bulky trifluoromethyl groups efficiently depressed the molecular packing and triplet-triplet annihilation (TTA) effect, and the low vibrational frequency of the C-F bond reduced the nonradiative transition rate. In addition, the nitrogen heterocycle also promoted the electron mobility of the Ir(III) complexes, thus enhancing the electron-hole injection balance. Therefore, the bipolar properties of the Ir(III) complexes are beneficial for their OLED performance with reduced efficiency roll-off [3].

## 2. Results and Discussion

### 2.1. Preparation and Characterization

**Scheme 1:**
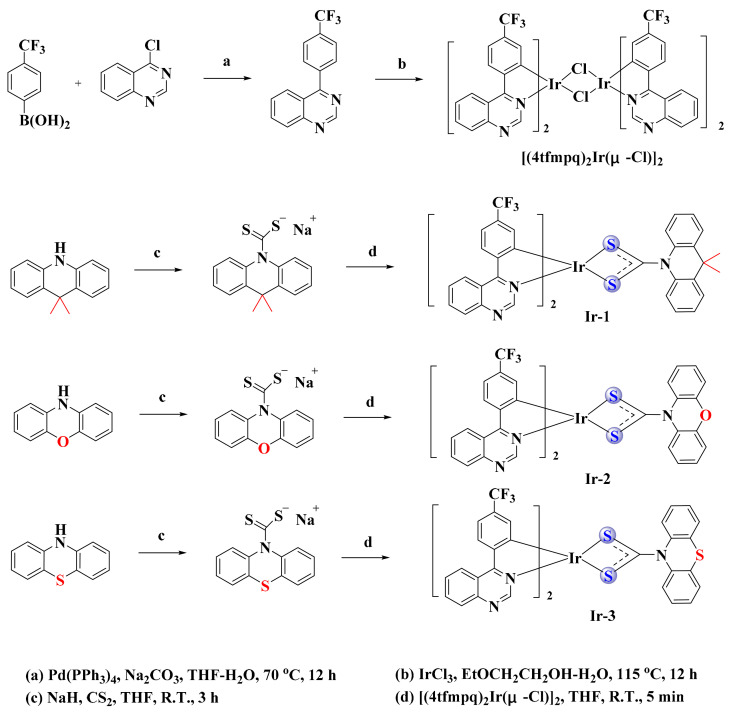
Synthesis routes of the ligands and Ir(III) complexes.

The chemical structures and synthetic routes of three Ir(III) complexes are showed in Scheme 1. The synthesized main ligand 4tfmpq via a Suzuki coupling reaction reacted with IrCl_3_·nH_2_O to obtain the [(4tfmpq)_2_Ir(μ-Cl)]_2_ dimer. Three S atom-containing ligand salts were synthesized by the reaction of 9,10-dihydro-9,9-dimethylacridine, phenoxazine, or phenothiazine with NaH and CS_2_. Due to the sensitivity to water and oxygen, the ancillary ligands were used directly without further purification [23]. Remarkably, all the Ir(III) complexes were prepared under quite mild conditions (5 min at room temperature in tetrahydrofuran) in yields of 48.9–85.5%, owing to the strong coordination capability of sulfur atoms with iridium atoms [24]. All these complexes were characterized by ^1^H NMR, ^13^C NMR, ^9^F NMR, and high-resolution mass spectrometer (HR-MS). Particularly, the single crystals of Ir-2 and Ir-3 were obtained by growing slowly through solvent diffusion from methanol into dichloromethane solutions at room temperature.

**Figure 1:**
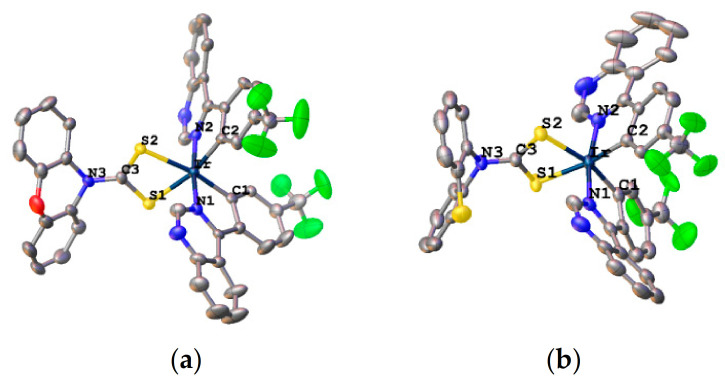
ORTEP diagrams of (**a**) Ir-2 (CCDC no. 2065084) and (**b**) Ir-3 (CCDC no. 2065079) with the atom-numbering schemes. Hydrogen atoms are omitted for clarity.

The single-crystal structures of Ir-2 and Ir-3 were further proved by X-ray crystallography (Figure 1), and the crystallographic data and the selected important bond lengths/angles are summarized in Tables S1 and S2, respectively. It is obvious that the metal iridium atom center exhibited a distorted three-dimensional octahedral coordination geometry with an S^S ancillary ligand and two C^N cyclometalated ligands. Both the phenothiazine ring and phenoxazine ring showed a nonplanar butterfly conformation, whereas the phenothiazine group formed a larger bend with the Ir-S-C-S plane than with the phenoxazine group. The quinazoline core framework showed a certain angle related to the adjacent 4-trifluoromethyl-phenyl unit, which was due to the steric hindrance originating from the through-space repulsion between the two nearby hydrogen atoms [25]. The similarity of bond lengths of S1–O3 (1.716(7) Å for Ir-2, 1.699(4) Å for Ir-3) and S2–O3 (1.708(7) Å for Ir-2, 1.706(4)Å for Ir-3) indicates that the −1 charge of the dithiocarbamate was delocalized over both sulfur atoms [26]. The four-membered Ir-S-C-S backbones led to significantly more acute S–Ir–S bite angles (71.35(5)° for Ir-2, 71.62(4)° for Ir-3) than those of the larger five- or six-membered heterocycles [13,27,28,29]. The Ir–S bond lengths (2.4486(17)–2.4643(12) Å) in an ancillary ligand were significantly longer than the Ir–C bond lengths (2.000(4)–2.020(5) Å) and the Ir–N bonds lengths (2.037(4)–2.053(4) Å) in cyclometalated ligands. All other bond lengths and angles were within the normal values.

Thermogravimetric analysis (TGA) measurements were carried out to investigate the thermal properties of these three Ir(III) complexes because the thermal stability of the emitters is vital for stable OLEDs. As shown in Figure S1 and Table 1, the decomposition temperatures (*T*_d_, 5% weight loss) of these Ir(III) complexes were 329, 343, and 375 °C for Ir-1, Ir-2, and Ir-3, respectively, indicating that all complexes had good thermal stability.

### 2.2. Photophysical Property

**Figure 2:**
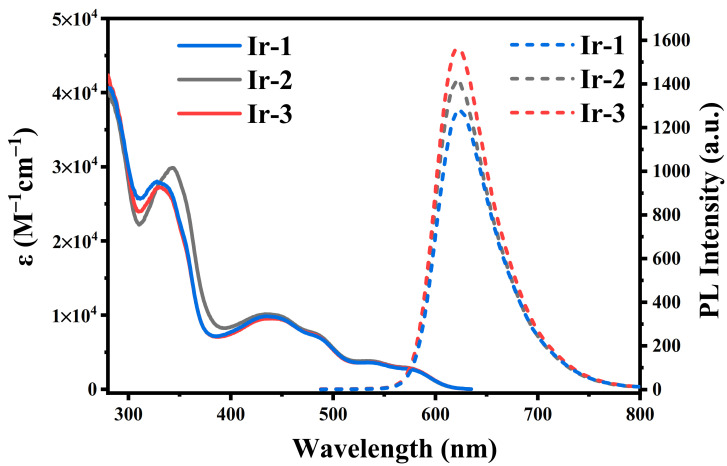
UV−vis absorption and PL spectra at 298 K of Ir-1, Ir-2, and Ir-3 complexes in dichloromethane (concentration: 10^−5^ M).

The ultraviolet-visible absorption and PL spectra of Ir-1, Ir-2, and Ir-3 recorded in dichloromethane solutions (10^−5^ M) at room temperature are shown in Figure 2, and the pertinent data are displayed in Table 1. The 3D correlation spectra of excitation and emission spectra of three complexes are illustrated in Figure S2. The intense absorption bands of the three complexes below 390 nm were attributed to spin-allowed π−π* transitions related to the ligands and the relatively weaker absorption parts in the region of 390–515 nm were assigned to ^1^MLCT. The lowest-lying absorption from 515 to 630 nm were ascribed to spin-forbidden ^3^MLCT and ^3^LC due to the strong spin-orbit coupling of the iridium atom [7,10,30].

Three Ir(III) complexes exhibited approximative red emission peaks with the maximum at 622, 619, and 622 nm for Ir-1, Ir-2, and Ir-3, respectively. The PL spectra measured at 298 K were broad and featureless, and those measured at 77 K (Figure S3) also did not show fine vibronic progressions, perhaps indicating the main emissions of these complexes were likely dominated by the ^3^MLCT [31,32]. The PL quantum yields of the three Ir(III) complexes were measured in degassed dichloromethane as 35.4%, 50.4%, and 52.8%, respectively (Table 1 and Figure S4), which were consist with their PL intensity sequence (Figure 2). As is known, the longer phosphorescent lifetime (*τ*) of the materials usually leads to a severer TTA both in PL and electroluminescence (EL), so materials with short *τ* values are favorable for fabrication of efficient OLEDs with low efficiency roll-off [33]. In this case, the three Ir(III) complexes showed relatively short *τ* values of 0.45, 0.46, and 0.59 μs (Table 1 and Figure S5), which was expected to increase spin-state mixing and delay the EL efficiency roll-off [34].

### 2.3. Electrochemical Property and Theoretical Calculation

The characteristics of frontier orbitals and the resulting highest occupied molecular orbital (HOMO) and the lowest unoccupied molecular orbital (LUMO) energy levels (*E*_HOMO_/*E*_LUMO_) are important to the electrochemical property of Ir(III) complexes, which can provide a theoretical basis for the design and fabrication of OLEDs [35]. Cyclic voltammetry (CV) measurements in deaerated CH_3_CN employing ferrocenium/ferrocene (Fc^+^/Fc) as the internal standard were carried out to study the redox properties and HOMO/LUMO levels of all Ir(III) complexes (Figure S6). However, the reduction behaviors of Ir(III) complexes were irreversible, so we were unable to estimate their HOMO and LUMO energies accurately [36]. According to the equations *E*_HOMO_ = −(*E*_ox_ − *E*_Fc/Fc_^+^ + 4.8) eV, *E*_gap_ *=* 1240/λ_abs-onset_, and *E*_LUMO_ = *E*_HOMO_ + *E*_gap_, the HOMO levels for Ir(III) complexes were estimated on the basis of an oxidation potential of 4.8 eV (below vacuum level) for Fc^+^/Fc, the LUMO levels were estimated on the sum of HOMO levels, and the energy band gap was determined by absorption edge (MLCT) in the absorption spectrum [20,37,38]. The ^3^MLCT absorption bands of the three iridium complexes reached the absorption edge and thus mainly participated in the electron semiconductivity [39], which was in agreement with the calculation results of the frontier molecular orbital distributions and energy splitting of singlet and triplet states of Ir-3 (Table S3). All complexes had similar *E*_HOMO_ and *E*_LUMO_ values (−5.67/−3.55, −5.57/−3.54, and −5.57/−3.56 eV, Table 1), which can be attributed to their similar structures.

**Figure 3:**
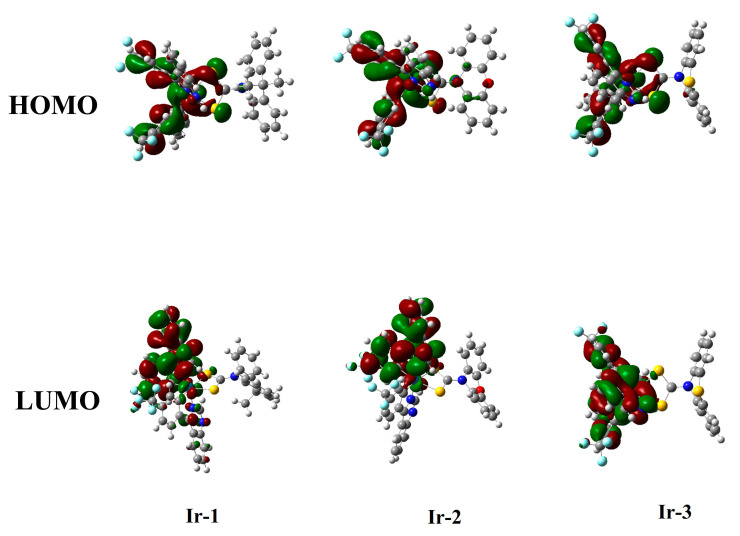
Theoretical HOMO and LUMO orbital electron cloud distributions of the three Ir(III) complexes.

To gain further insight into the electronic properties of the three complexes, density functional theory (DFT) calculations utilizing the Gaussian09 software were performed and the theoretical HOMO/LUMO orbital distributions of Ir-1, Ir-2, and Ir-3 are listed in Figure 3. The corresponding HOMO and LUMO electron cloud density distributions of each fragment of the Ir(III) complexes are listed in Table S4, which reveals that the HOMO and LUMO levels of Ir-1, Ir-2, and Ir-3 had almost identical distributions in iridium atoms, main ligands, and ancillary ligands. As shown in Figure 3 and Table S4, the HOMO orbitals of the Ir(III) complexes were mostly located on the main ligand (47.2–52.1%) together with the d-orbitals of the iridium atoms (36.7–40.0%) with a small portion of the ancillary ligands (10.8–12.8%). The LUMO orbitals were mostly distributed over the π* orbitals of the main ligands (94.7–95.1%) and a small extent on the d-orbitals of the iridium (2.7–3.9%) and ancillary ligands (1.3–2.6%). These results suggest that the ancillary ligands contributed much less in HOMO and LUMO distributions than the main ligands, so the difference of ancillary ligands had little impact on the photophysical properties of the three Ir(III) complexes.

### 2.4. OLED Performance

**Scheme 2:**
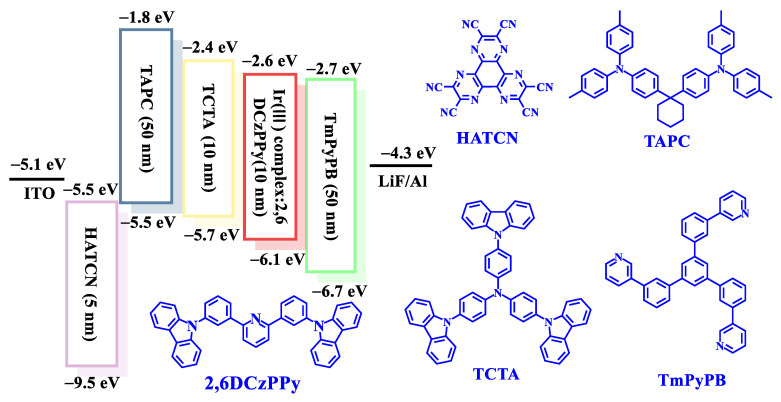
Energy level diagram of the HOMO and LUMO levels of materials investigated and their chemical molecular structures.

**Figure 4:**
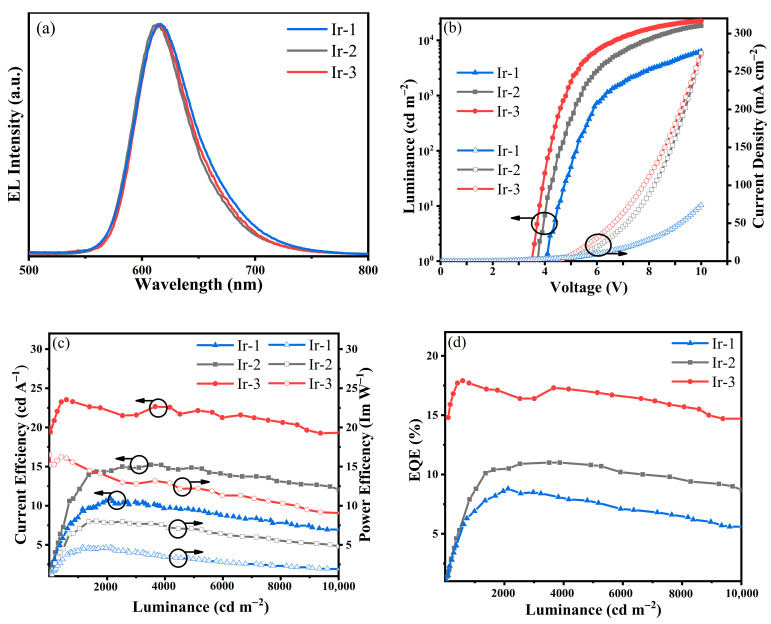
Electroluminescence performances of D1, D2, and D3: (**a**) electroluminescence spectra measured in 6 V, (**b**) current density–voltage curves and luminance–voltage curves, (**c**) current efficiency–luminance curves and power efficiency–luminance curves, and (**d**) EQE–luminance curves.

To illustrate the EL properties of these complexes, the single emitting layer (EML) devices named D1, D2, and D3 using Ir-1, Ir-2, and Ir-3 as emitters, respectively, were fabricated with the configuration of ITO (indium tin oxide)/HAT-CN (dipyrazino[2,3-f,2′,3′-h]quinoxaline-2,3,6,7,10,11-hexacarbonitrile, 5 nm)/TAPC (4,4′-cyclohexylidenebis[*N*,*N*-bis-(4-methylphenyl)aniline], 40 nm)/TCTA (4,4″,4″-tris(carbazol-9-yl)triphenylamine, 10 nm)/Ir(III) complex (10 wt%): 2,6DCzPPy (2,6-bis-(3-(carbazol-9-yl)phenyl)pyridine, 10 nm)/TmPyPB (1,3,5-tri(mpyrid-3-yl-phenyl)benzene, 50 nm)/LiF (1 nm)/Al (100 nm). As shown in Scheme 2, the HAT-CN, LiF, TAPC, and TmPyPB materials served as hole-injecting, electron-injecting, hole-transport, and electron-transport interface modified materials, respectively. The TCTA material also served as an electron-blocking layer material. The proper HOMO and LUMO energy levels of TCTA can not only match the adjacent carrier transport materials to reduce carrier injection and transport barriers, but also lower driving voltage. To reduce the TTA effect and prevent exciton diffusion and quenching in the adjacent layers of materials in OLEDs, the Ir(III) complexes have to be dispersed into the suitable host matrix [4,40]. Moreover, considering energy level matching in the host-guest system, the bipolar material 2,6DCzPPy with good and balance carrier transport proper acts as the host material for doping concentrations of Ir(III) emitters so that energy can transfer efficiently in the emitting layer (EML). The overall electroluminescence performances are shown in Figure 4 and Table 2.

The EL emission peaks of the D1, D2, and D3 devices were 612, 613, and 619 nm, with CIE coordinates at (0.62, 0.34), (0.64, 0.35), and (0.63, 0.35), respectively, which were very close to those measured in CH_2_Cl_2_ solutions, indicating that the EL emissions of the devices originated from the Ir(III) complexes. No obvious host material emissions were observed in the three devices, suggesting that the energy transferred efficiently from the host material to the Ir(III) complexes in EML.

Owing to the same main ligand and similar ancillary ligand structures of the three Ir(III) complexes, the device performances mainly depended on their PL efficiencies. Respectively, device D1, employing Ir-1 (Φ_P_ is 35.4%), showed the lowest EL performance, with a maximum luminance (*L*_max_) of 14,080 cd A^−1^, a *η*_c,max_ of 10.86 cd A^−1^, a maximum power efficiency (*η*_p,max_) of 4.70 lm W^−1^, and an EQE_max_ of 8.8%. Comparatively, device D2, based on Ir-2 (Φ_P_ is 50.4%), exhibited better characteristics, with an *L*_max_, *η*_c,max_, *η*_p,max_, and EQE_max_ of 18,740 cd m^−2^, 15.23 cd A^−1^, 8.04 lm W^−1^, and 11.0%, respectively. Due to the highest Φ_P_ of Ir-3 (52.8%), device D3 achieved the best EL performance, with an *L*_max_ of 22,480 cd m^−2^, an *η*_c,max_ of 23.71 cd A^−1^, an *η*_p,max_ of 16.23 lm W^−1^,n and an EQE_max_ of 18.1%, respectively.

Summarized from the investigated results, all devices achieved good OLED properties. The possible reasons were summarized as follows: Firstly, different electron-donating moieties in dithiocarbamate derivatives and the nitrogen frame of cyclometalated ligand equipped the Ir(III) complexes with bipolar properties, which may have helped to widen the recombination area and balance distribution for the excitons. Secondly, the sulfur atom had strong coordination capability with the iridium atom so they could form stable dithiolate compounds that could reduce the work function of the emitter and the threshold electric field [20]. Lastly, the bulky nonplanar butterfly conformation of the ancillary ligands sufficiently inhibited molecular aggregation and the formation of intermolecular excimers, consequently reducing the intermolecular interactions [8]. Compared to the previously reported Ir(III) complexes with sulfur-containing four-membered ancillary ligands (Table S5), the performances of the three Ir(III) complexes were not excellent due to the moderate PLQYs of three emitters.

## 3. Experimental Section

### 3.1. General Procedures

All reagents and chemicals were purchased from commercial sources and used without further purification. ^1^H NMR, ^13^C NMR and ^19^F NMR spectra were measured on a Bruker AM 400 spectrometer (Bruker, Karlsruhe, Germany). High-resolution electrospray mass spectra (HR-MS) were measured on a MTQ III q-TOF (Bruker, Karlsruhe, Germany). Absorption and photoluminescence spectra were measured on a UV-3100 spectrophotometer (Shimadzu, Kyoto, Japan) and a Hitachi F-4600 photoluminescence spectrophotometer (Hitachi, Kyoto, Japan), respectively. TG-DSC measurements were carried out on a TG sf/1100 (Mettler Toledo, Zurich, Switzerland). Cyclic voltammetry measurements were conducted on a MPI-A multifunctional electrochemical and chemiluminescent system (CH Instruments, TX, USA) at room temperature with a polished Pt plate as the working electrode, a platinum thread as the counter electrode and a Ag-AgNO_3_ (0.1 M) in CH_3_CN as the reference electrode, tetra-nbutylammonium perchlorate (0.1 M) was used as the supporting electrolyte, using Fc^+^/Fc as the internal standard, the scan rate was 0.1 V/s. The absolute photoluminescence quantum yields and the decay lifetimes of the compounds were measured with HORIBA FL-3 fluorescence spectrometer(HORIBA, Kyoto, Japan). Thermogravimetric analysis (TGA) was performed on a TG sf/1100 under nitrogen at a heating rate of 10 °C min^−1^.

### 3.2. Synthesis of Ligands and Complexes

#### 3.2.1. Synthesis of the Main Ligand and [(C^N)_2_Ir(μ-Cl)]_2_ Chloride-Bridged Dimer

4-Chloro-quinazoline (6 mmol), 4-(trifluoromethyl)phenylboronic acid (5 mmol), Na_2_CO_3_ (10 mmol), and Pd(PPh_3_)_4_ (0.1 mmol) were added to 40 mL THF/H_2_O (3:1, *v*/*v*) and the mixture was refluxed at 75 °C for 12 h. The organic phase was extracted with CH_2_Cl_2_ and purified by silica gel column chromatography (CH_2_Cl_2_/petroleum ether 1:2 (*v*/*v*)). A total of 1.24 g main ligand 4tfmpq as white powder was obtained with a yield of 75.6%.

IrCl_3_ (2.14 mmol) and 4tfmpq (5.14 mmol) were added to a 2-ethoxyethanol and water mixture. Then the solution was heated at 110 °C for 12 h. After the addition of water, the precipitated red powder of [(4tfmpq)_2_Ir(μ-Cl)]_2_ chloride-bridged dimer was filtered, washed, and dried in vacuum with a yield of 84.7% (2.8 g).

#### 3.2.2. General Synthesis of the Ancillary Ligands and Ir(III) Complexes

NaH (2.2 mmol) was added to a 10 mL ultra-dry tetrahydrofuran solution of 9,10-dihydro-9,9-dimethylacridine, phenoxazine, or phenothiazine (2 mmol) in an ice-water bath under an N_2_ atmosphere. After stirring for 1 h at room temperature, CS_2_ (2.2 mmol) was added to the system and the solution was stirred for another 2 h. The sodium thionocarboxylate aqueous solutions were used in the next procedure without further purification.

#### 3.2.3. General Synthesis of the Ir(III) Complexes

The [(4tfmpq)_2_Ir(μ-Cl)]_2_ dimer (0.83 mmol) was added to the sodium thionocarboxylate aqueous solutions and reacted for 5 min at room temperature. Then the solutions were concentrated and the resulting residues were purified by silica gel column chromatography (CH_2_Cl_2_/petroleum ether 2:1 (*v*/*v*)) to obtain the final Ir(III) complexes.

Ir-1. Yield: 62.7%. ^1^H NMR (400 MHz, CDCl_3_) δ 10.26 (s, 2H), 8.81 (s, 2H), 8.40. (d, *J* = 6.6 Hz, 2H), 8.26 (s, 2H), 8.08–7.71 (m, 6H), 7.45 (s, 2H), 7.22 (d, *J* = 6.8 Hz, 2H), 7.18 (s, 2H), 7.16 (s, 2H), 6.75 (s, 2H), 1.81 (s, 2H), 1.61 (s, 2H), 1.35 (s, 2H). ^13^C NMR (101 MHz, CDCl_3_) δ 213.24, 173.23, 162.18, 154.17, 149.42, 145.91, 141.18, 136.97, 133.63, 130.76, 130.33, 130.02, 128.23, 127.97–127.30 (m), 126.34, 125.22, 124.77, 124.34, 123.05, 117.61–116.90 (m), 37.32. HR-MS Calculated: 1024.1549 for C_46_H_30_F_6_IrN_5_S_2_ [M + H]^+^, found: 1024.1551.

Ir-2. Yield: 48.9%. ^1^H NMR (400 MHz, CDCl_3_) δ 10.26 (s, 2H), 8.82 (d, *J*= 8.5 Hz, 2H) 8.41 (d, *J* = 8.4 Hz, 2H), 8.28 (d, *J* = 8.2 Hz, 2H), 8.04–7.98 (m, 2H), 7.94 (d, *J* = 7.7 Hz, 2H), 7.88–7.82 (m, 2H), 7.25–7.16 (m, 6H), 7.05 (ddd, *J* = 8.6, 6.6, 2.2 Hz, 2H), 6.71 (s, 2H). 13C NMR (101 MHz, CDCl_3_) δ 215.89, 174.25, 162.74, 155.15, 150.46 (d, *J* = 4.6 Hz), 146.89, 134.80, 131.82, 131.50, 131.19, 129.32, 129.10–128.98 (m), 128.98–128.17 (m), 128.15 (d, *J* = 2.3 Hz), 126.03, 125.77, 124.76, 122.49, 121.89, 118.37 (dd, *J* = 7.0, 3.5 Hz), 117.65. HR-MS Calculated: 998.1029 for C_43_H_24_F_6_IrN_5_OS_2_ [M + H]^+^, found: 998.1026.

Ir-3. Yield: 85.5%. ^1^H NMR (400 MHz, CDCl_3_) 10.51 (s, 1H), 9.96 (s, 1H), 8.81 (m, 2H), 8.34 (m, 4H), 8.13–7.71 (m, 5H), 7.69–7.37 (m, 3H), 7.23 (m, 6H), 6.67 (m, 2H). ^13^C NMR (101 MHz, CDCl_3_) δ 216.8, 174.2, 150.5, 146.9, 137.6, 134.7, 132.9, 131.7, 131.3 (q, *J* = 30.9 Hz), 129.3, 128.8 (d, *J* = 3.1 Hz), 128.6 (q, *J* = 4.0 Hz), 128.6, 128.0 (d, *J* = 11.7 Hz), 127.8, 126.3 (d, *J* = 4.6 Hz), 125.8, 121.9, 118.3 (q, *J* = 3.9 Hz). HR-MS Calculated: 1014.0800 for C_43_H_24_F_6_IrN_5_S_3_ [M + H]^+^, found: 1014.0800.

### 3.3. OLED Fabrication and Measurement

All OLEDs were fabricated on the pre-patterned ITO-coated glass substrate with a sheet resistance of 15 Ω/sq. The deposition rate for the organic compounds was 1–2 Å/s. The phosphor and the host 2,6DCzPPy were co-evaporated to form an emitting layer from two separate sources. The cathode consisting of LiF/Al was deposited by evaporation of LiF with a deposition rate of 0.1 Å/s and then by evaporation of Al metal with a rate of 3 Å/s. The effective area of the emitting diode was 0.1 cm^2^. The thickness of each layer was calculated by the readings of crystal oscillators. The brightness of the device was recorded with a luminance meter (ST-86LA, Beijing Normal University Photoelectric Instrument Factory) and the current density was detected by Keithley 2400 (Tektronix Inc) with experimental uncertainties of about 5%.

## 4. Conclusions

In summary, three red Ir(III) complexes, Ir-1, Ir-2, and Ir-3 with four-membered ring Ir-S-C-S structures were obtained rapidly at room temperature in 5 min with the nitrogen heterocycle 4tfmpq as the main ligand and different thionocarboxylates as the ancillary ligands. The diversity of the four-membered ring ligands was greatly enriched by employing different electron-donating groups 9,10-dihydro-9,9-dimethylacridine, phenoxazine, and phenothiazine in the ancillary ligands. Due to the same main ligand and similar ancillary ligand structures, the three Ir(III) complexes bore similar emission wavelengths at around 619 nm with phosphorescence quantum yields from 35.4% to 52.8% and short excited lifetimes lower than 1 μs. With the three complexes as dopants, all devices achieved good performances. However, the device using the Ir(III) complex with phenothiazine as an electron-donating group in the ancillary ligand displayed the best performances, with an *L*_max_ of 22,480 cd m^−2^, an *η*_c,max_ of 23.71 cd A^−1^, and an EQE_max_ of 18.1%.

## Figures and Tables

**Table 1:** Physical and photophysical properties of the three complexes.

Complex	*T*_d_ ^a^ (°C)	Absorption ^b^ (λ, nm)	Emission ^b^ (λ_max_, nm)	*τ*_298K_ ^c^ (μs)	Φ_P_ ^c^ (%)	HOMO/LUMO ^d^ (eV)
Ir-1	329	326/437	622	0.45	35.4	−5.67/−3.55
Ir-2	343	341/433	619	0.46	50.4	−5.57/−3.54
Ir-3	375	328/437	622	0.59	52.8	−5.57/−3.56

^a^ Decomposition temperature. ^b^ Measured in DCM (10^−5^ M) at 298 K. ^c^ Measured in DCM (10^−5^ M) in a N_2_ atmosphere. ^d^ HOMO energy levels calculated from cyclic voltammetry analysis. LUMO energy levels calculated from cyclic voltammetry analysis and UV−Vis onset.

**Table 2:** The key EL data of devices D1–D3.

Device	*V*_turn-on_ ^a^ (V)	*L*_max_ ^b^ [cd m^−2^(V)]	*η*_c,max_ ^c^ [cd A^−1^(V)]	*η*_p,max_ ^d^ [lm W^−1^(V)]	EQE_max_ ^e^ (%)	CIE (x,y)
D1	4.1	14,080(13.3)	10.86(7.3)	4.70(6.4)	8.8	(0.62, 0.34)
D2	3.8	18,740(10.3)	15.23(6.2)	8.04(5.6)	11.0	(0.64, 0.35)
D3	3.5	22,480(10.2)	23.71(5.1)	16.23(4.5)	18.1	(0.63, 0.35)

^a^ Turn-on voltage at 1 cd m^−2^. ^b^ Maximum luminance. ^c^ Maximum current efficiency. ^d^ Maximum power efficiency. ^e^ Maximum external quantum efficiency calculated within visible spectrum region.

## Data Availability

The data presented in this study are available in supplementary material.

## Supplementary Materials

The following are available online. Figure S1: The TG curve of the three Ir(III) complexes under nitrogen at a heating rate of 10 °C min^−1^; Figure S2: 3D excitation–emission correlation spectrums of Ir-1, Ir-2, and Ir-3; Figure S3: PL spectra at 77 K of Ir-1, Ir-2, and Ir-3 complexes in dichloromethane solutions (10^−5^ M); Figure S4: PLQY of Ir-1, Ir-2, and Ir-3 in degassed dichloromethane solution (10^−5^ M); Figure S5: The lifetime curves of Ir-1, Ir-2, and Ir-3 in degassed dichloromethane solution (10^−5^ M); Figure S6: CV curves of the Fc, Ir-1, Ir-2, and Ir-3 in deaerated CH_3_CN solution (10^−5^ M) Figure S7: ^1^H NMR spectrum of Ir-1; Figure S8: ^1^H NMR spectrum of Ir-2; Figure S9: ^1^H NMR spectrum of Ir-3; Figure S10: ^13^C NMR spectrum of Ir-1; Figure S11: ^13^C NMR spectrum of Ir-2; Figure S12: ^13^C NMR spectrum of Ir-3; Figure S13: ^19^F NMR spectrum of Ir-1; Figure S14: ^19^F NMR spectrum of Ir-2; Figure S15: ^19^F NMR spectrum of Ir-3; Table S1: The crystallographic data of Ir-2 and Ir-3; Table S2: Selected bond lengths and angles of Ir-2 and Ir-3; Table S3: Frontier molecular orbital distributions and energy splitting of singlet and triplet states of Ir-3; Table S4: HOMO and LUMO electron cloud density distributions of each fragment of all Ir(III) complexes; Table S5: The reported device performances with Ir(III) complexes based on sulfur-containing four-membered ancillary ligands.

## Sample Availability

Samples of the compounds are not available from the authors.
