# Supplementary material for: Simple Synthesis of Red Iridium(III) Complexes with Sulfur-Contained Four-Membered Ancillary Ligands for OLEDs
Source: Molecules. 2021 Apr 29;26(9):2599. doi: 10.3390/molecules26092599 (PMC8125720; doi:10.3390/molecules26092599)
Supplement: Supplementary file 1 [file molecules-26-02599-s001.zip › molecules-1189334-supplementary.pdf]

Supplementary Materials

Simple Synthesis of Red Iridium(III) Complexes with Sulfur-Contained Four-Membered Ancillary Ligands for OLEDs

Meng-Xi Mao [1], Fang-Ling Li [1], Yan Shen [1], Qi-Ming Liu [1], Shuai Xing [1], Xu-Feng Luo [1], Zhen-Long Tu [1], Xue-Jun Wu [1] and You-Xuan Zheng [1,2,*]

[1] State Key Laboratory of Coordination Chemistry, Collaborative Innovation Center of Advanced Microstructures, Jiangsu Key Laboratory of Advanced Organic Materials, School of Chemistry and Chemical Engineering, Nanjing University, Nanjing 210023, China; mf1924021@smail.nju.edu.cn (M.-X.M.); mg1824034@smail.nju.edu.cn (F.-L.L.); mf1824030@smail.nju.edu.cn (Y.S.); 171850043@smail.nju.edu.cn (Q.-M.L.); mf20240035@smail.nju.edu.cn (S.X.); dg1924062@smail.nju.edu.cn (X.-F.L.); dg1824074@smail.nju.edu.cn (Z.-L.T.); xjwu@nju.edu.cn (X.-J.W.)

[2] Green Catalysis Center and College of Chemistry, Zhengzhou University, Zhengzhou 450001, China

* Correspondence: yxzheng@nju.edu.cn

Table S1. The crystallographic data of Ir-2 and Ir-3.

	Ir-2	Ir-3
Formula	4($C_{43}H_{24}F_6IrN_5OS_2$), 4(CH_2Cl_2), 4(CH_4O)	4($C_{43}H_{24}F_6IrN_5S_3$)
Formula weight	4455.83	4052.20
Temperature/K	193.01	193.01
Wavelength (Å)	1.34139	1.34139
Crystal system	monoclinic	monoclinic
Space group	$P2_1/c$	$P2_1/n$
a (Å)	20.5921(9)	12.417(2)
b (Å)	8.4426(3)	15.623(3)
c (Å)	24.3968(10)	19.760(4)
α (deg)	90	90.00(3)
β (deg)	104.224(2)	98.71(3)
γ (deg)	90	90.00(3)
V (Å^3)	4111.4(3)	3789.2(13)
Z	1	1
ρ_{calcd} (g/cm^3)	1.800	1.776
μ (Mo Kα) (mm^{-1})	6.182	6.134
F (000)	2192.0	1984
Range of transm factors(deg)	3.852–107.806	6.302–107.764
Reflns collected	24158	23356
Unique(Rint)	7440(0.0481)	6857 (0.0489)
Data/restraints/parameters	7440/6/578	6857/183/579
$R1^a$, wR_2^b[I > 2s(I)]	0.0476, 0.1243	0.0347, 0.0805
$R1^a$, wR_2^b (all data)	0.0610, 0.1324	0.0385, 0.0823
GOF on F^2	1.061	1.154
CCDC NO.	2065084	2065079

$R_1{}^a = \Sigma||F_o|-|F_c||/\Sigma|F_o|$. $wR_2{}^b = [\Sigma w(F_o{}^2 - F_c{}^2)^2/\Sigma w(F_o{}^2)]^{1/2}$

Table S2. Selected bond lengths and angles of Ir-2 and Ir-3.

	Ir-2	Ir-3
Selected Bonds	Bond length (Å)	Bond length (Å)

Ir-C(1)	2.007(6)	2.020(5)
Ir-C(2)	2.018(7)	2.000(4)
Ir-N(1)	2.037(5)	2.053(4)
Ir-N(2)	2.039(5)	2.037(4)
Ir-S(1)	2.4486(17)	2.4643(12)
Ir-S(2)	2.4486(17)	2.4591(13)
S(1)-C(3)	1.716(7)	1.699(4)
S(2)-C(3)	1.708(7)	1.706(4)
C(3)-N(3)	1.347(9)	1.363(5)
Selected angles	(°)	(°)
C(1)-Ir-N(1)	79.1(2)	78.94(17)
C(2)-Ir-N(2)	79.2(2)	79.11(15)
S(1)-Ir-S(2)	71.35(5)	71.62(4)
S(2)-C(3)-S(1)	113.3(4)	115.6(2)
C(3)-S(2)-Ir	87.6(2)	85.95(15)
C(3)-S(1)-Ir	87.7(2)	85.94(14)
N(3)-C(3)-S(1)	123.0(5)	122.5(3)

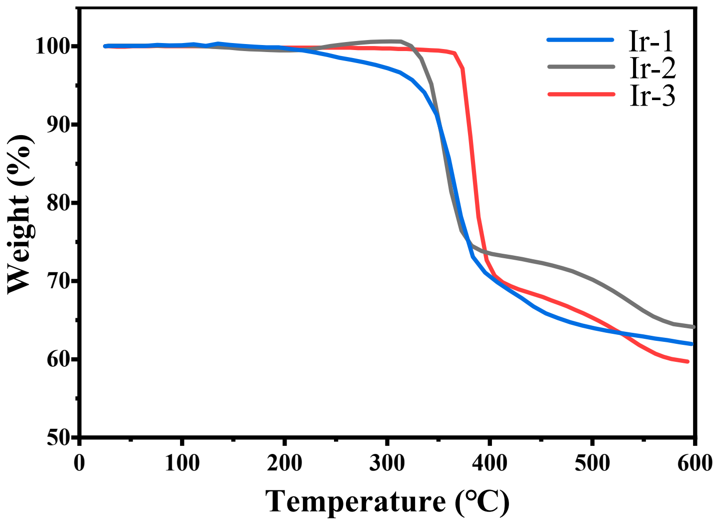

Figure S1. The TG curves of the Ir-1, Ir-2 and Ir-3 complexes under nitrogen at a heating rate of 10 °C min^{-1}.

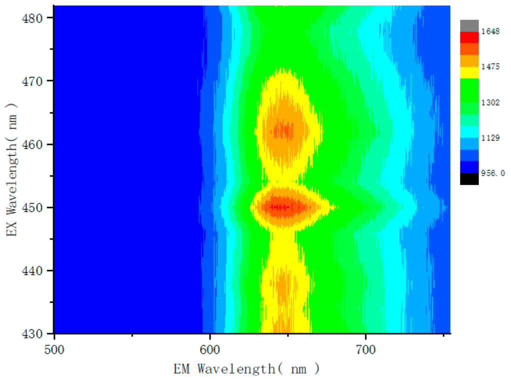

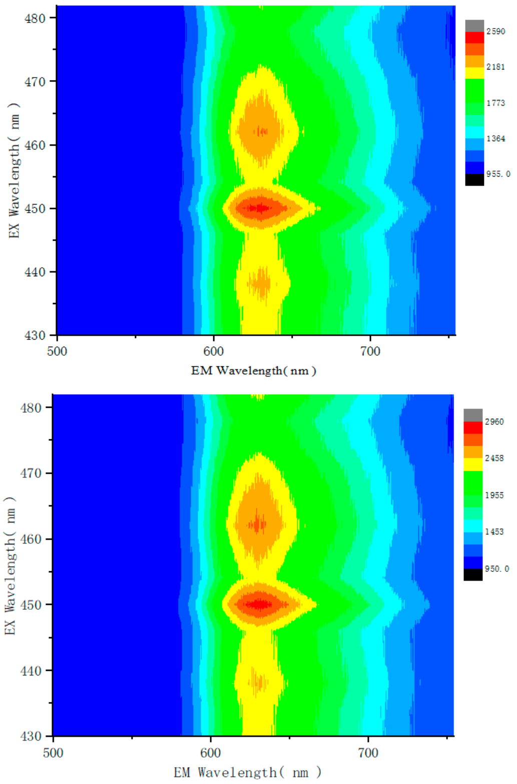

Figure S2. 3D excitation-emission correlation maps of the Ir-1, Ir-2 and Ir-3 complexes in degassed dichloromethane solutions (10^{-5} M).

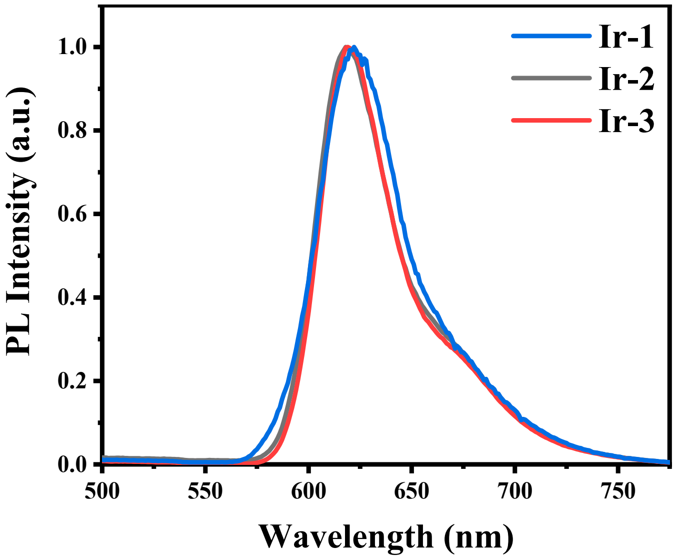

Figure S3. PL spectra at 77 K of Ir-1, Ir-2, and Ir-3 complexes in dichloromethane solutions (10^{-5} M).

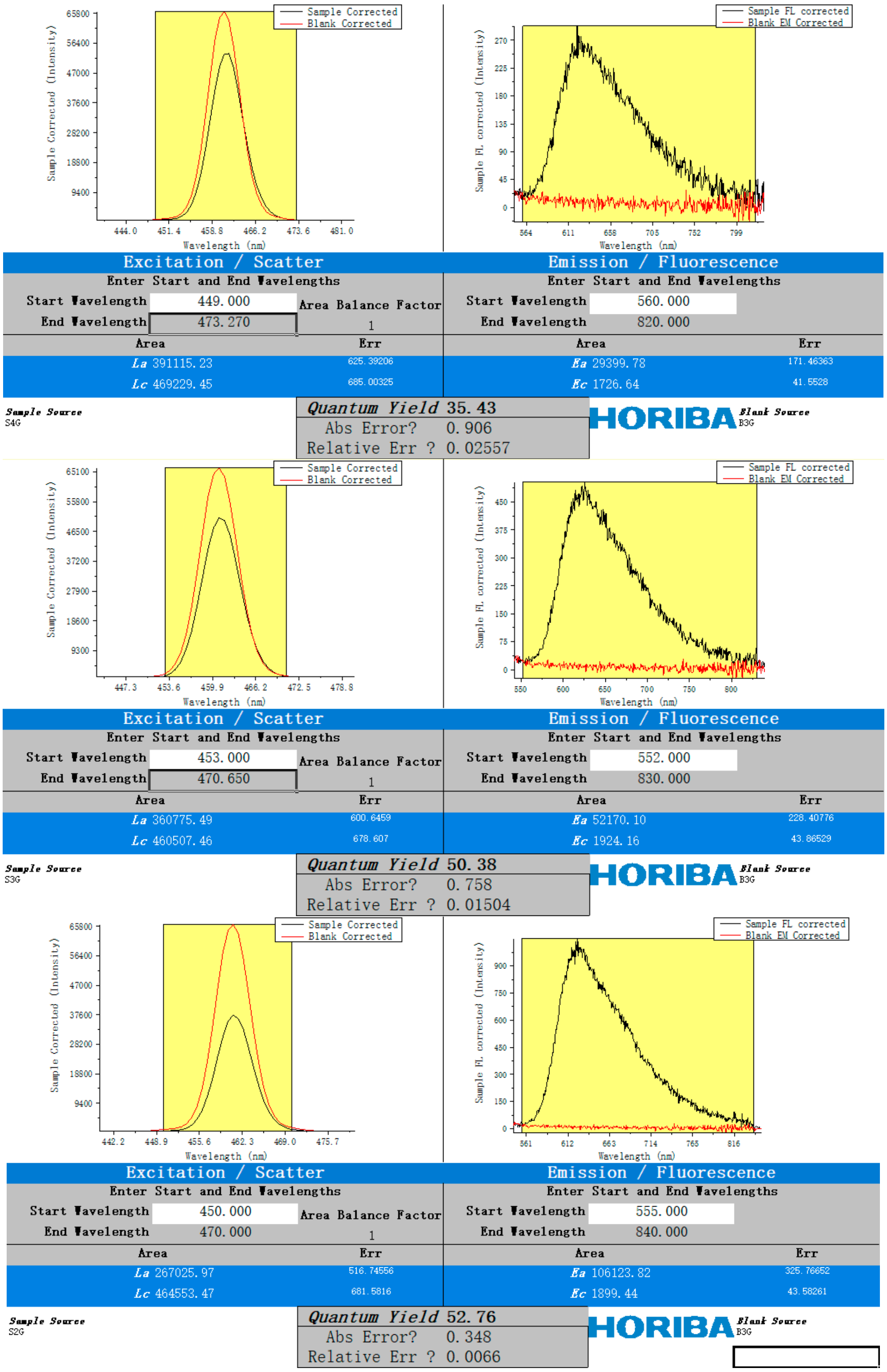

Figure S4. PLQYs of the Ir-1, Ir-2 and Ir-3 complexes in degassed dichloromethane solutions (10^{-5} M).

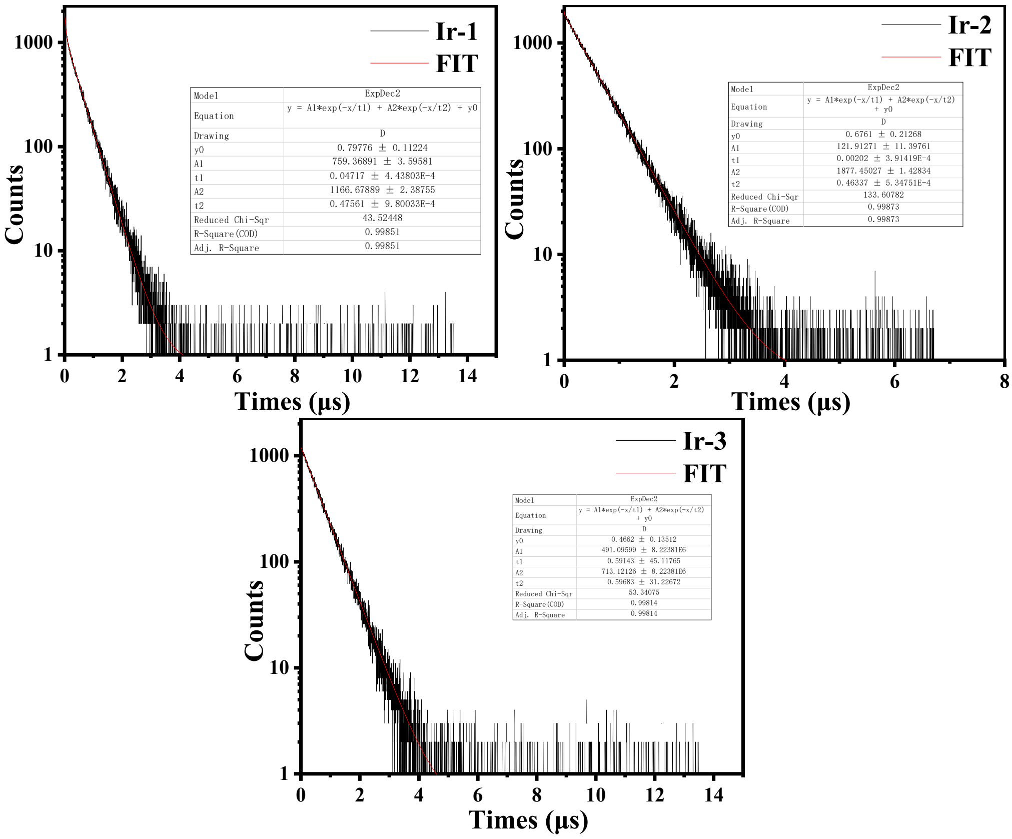

Figure S5. The lifetime curves of the Ir-1, Ir-2 and Ir-3 complexes in degassed dichloromethane solutions (10^{-5} M).

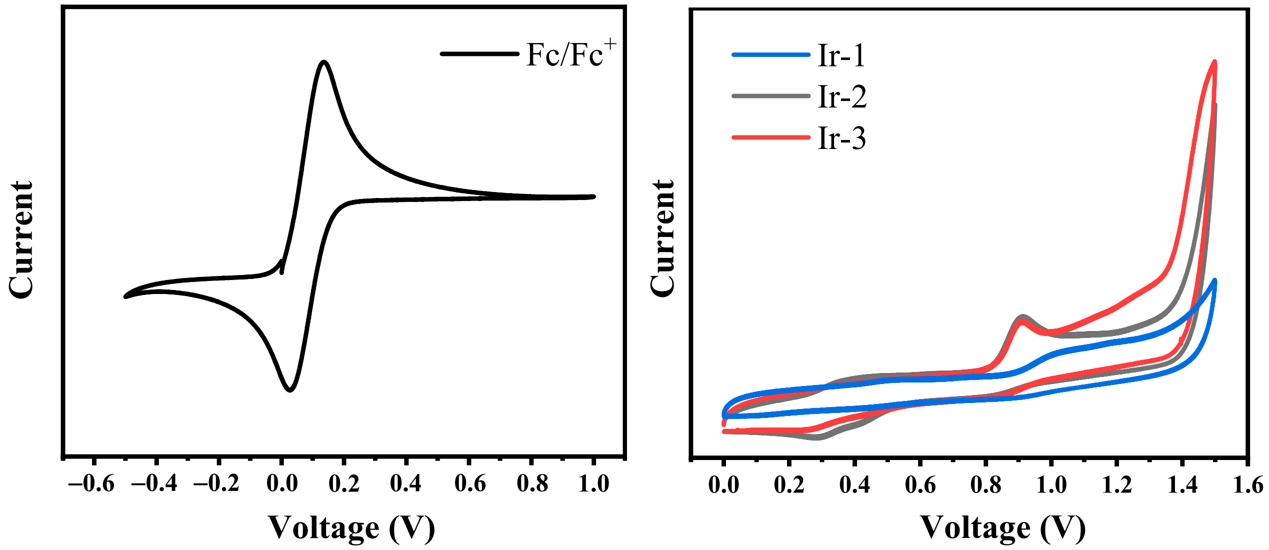

Figure S6. CV curves of the Fc, Ir-1, Ir-2 and Ir-3 complexes in deaerated CH_3CN solutions (10^{-5} M).

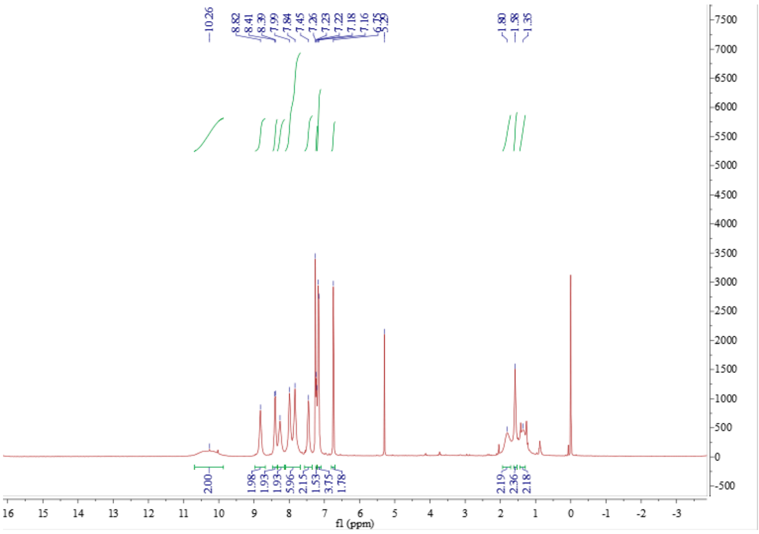

Figure S7. ^{1}H NMR spectrum of Ir-1.

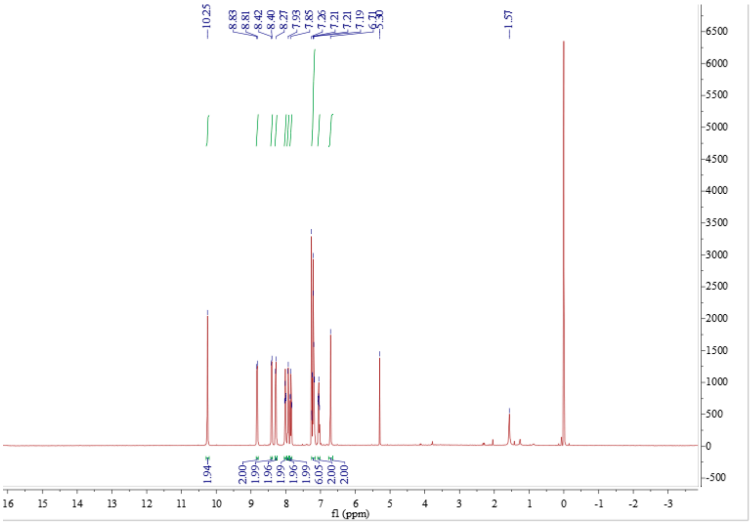

Figure S8. ^{1}H NMR spectrum of Ir-2.

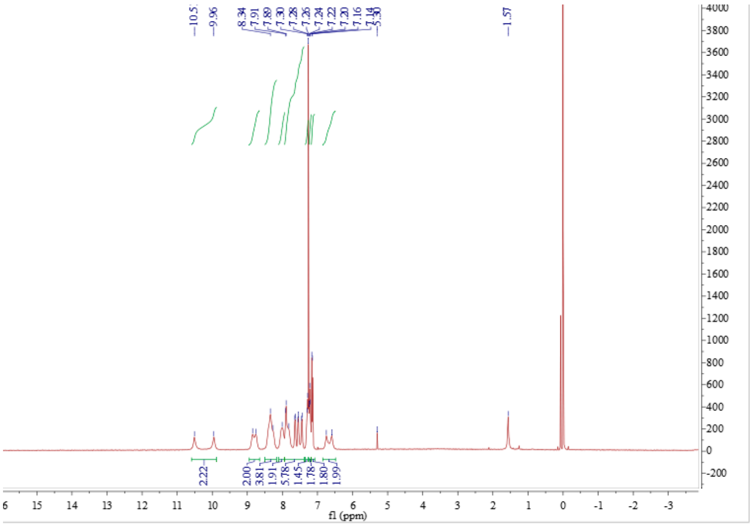

Figure S9. ^{1}H NMR spectrum of Ir-3.

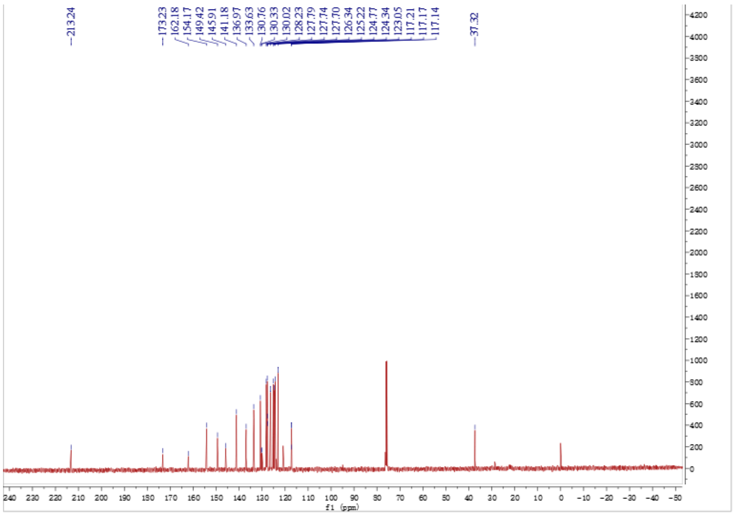

Figure S10. ^{13}C NMR spectrum of Ir-1.

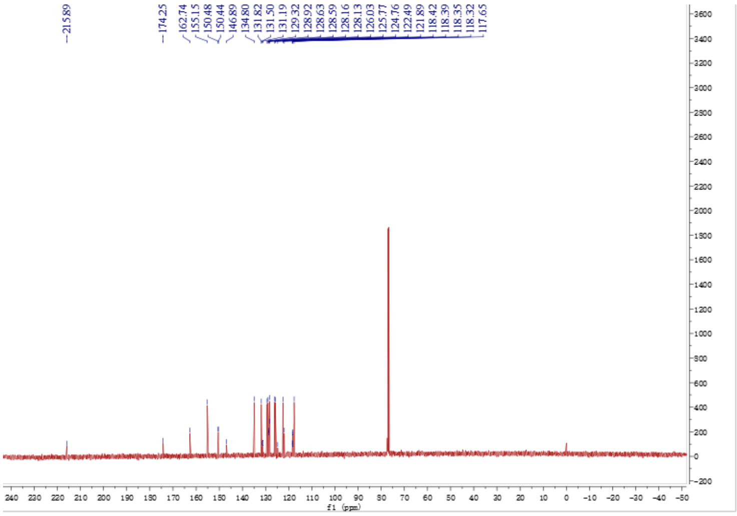

Figure S11. ^{13}C NMR spectrum of Ir-2.

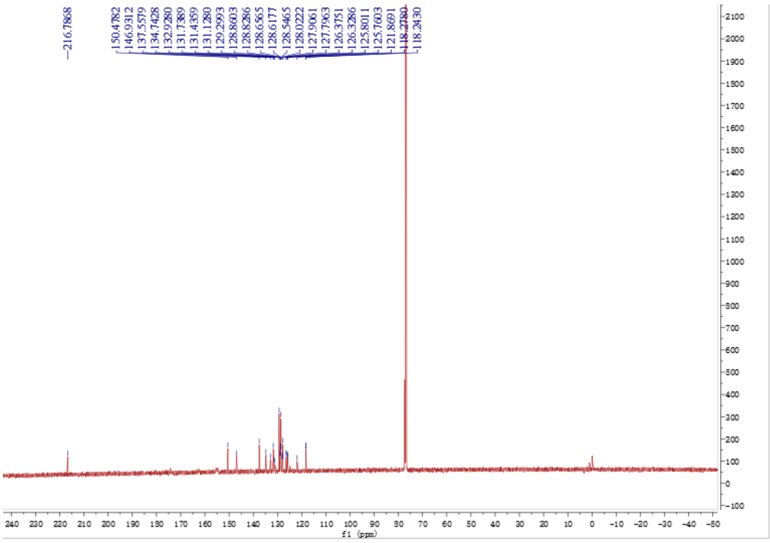

Figure S12. ^{13}C NMR spectrum of Ir-3.

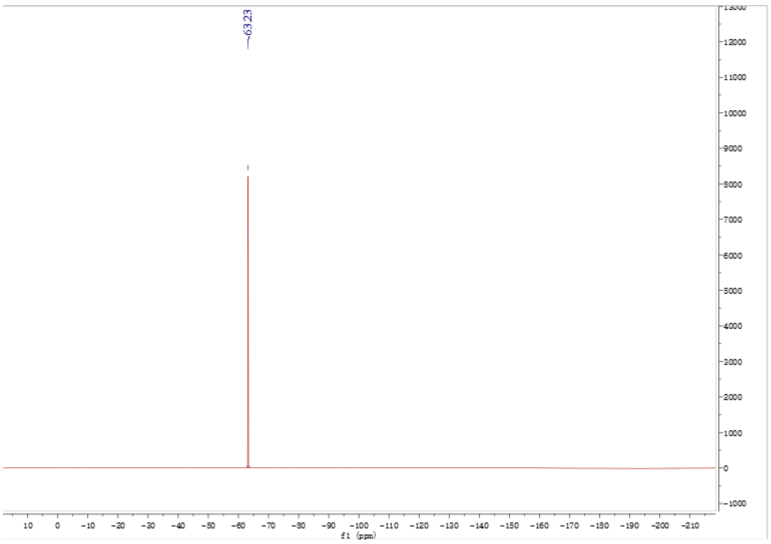

Figure S13. ^{19}F NMR spectrum of Ir-1.

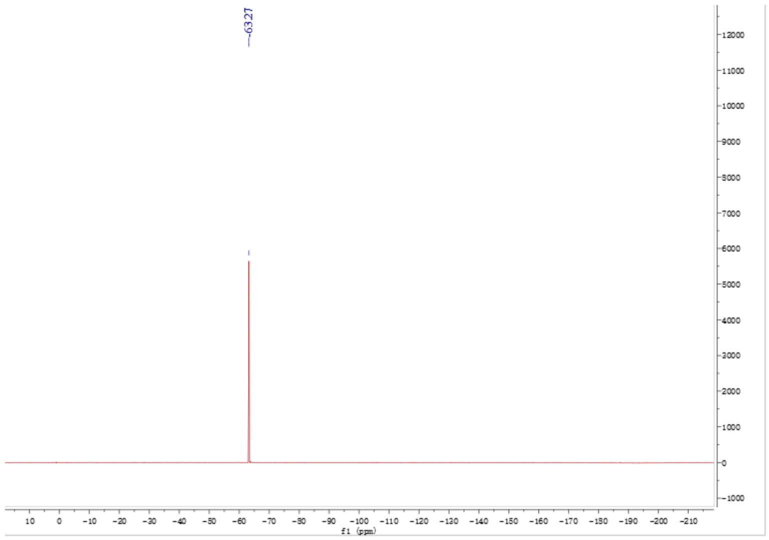

Figure S14. ^{19}F NMR spectrum of Ir-2.

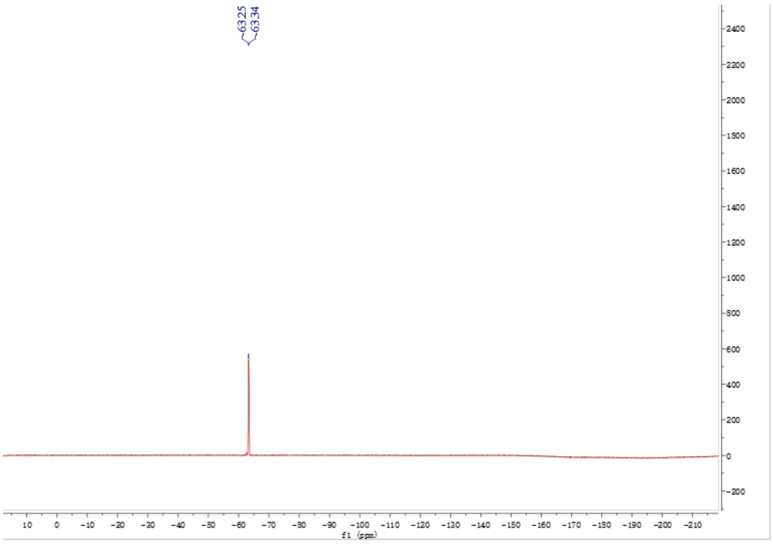

Figure S15. ^{19}F NMR spectrum of Ir-3.

Table S3. Frontier molecular orbital distributions and energy splitting of singlet and triplet states of Ir-3.

Frontier orbitals	HOMO	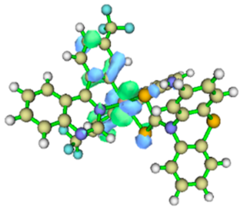
	LUMO	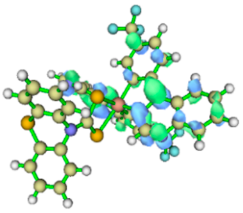
T_1 (eV)		2.1573 eV (HOMO→LUMO 83.8%)
S_1 (eV)		2.3790 eV (HOMO→LUMO 43.3%)

Table S4. HOMO and LUMO electron cloud density distributions of each fragment of all Ir(III) complexes.

**Complexes**	**Orbit**	**Composition(%)**		
		**Iridium**	**Main ligand**	**Ancillary ligand**
**Ir-1**	HOMO	40.0%	49.8%	10.8%
	LUMO	3.9%	94.9%	1.3%
**Ir-2**	HOMO	36.7%	52.1%	11.2%
	LUMO	3.3%	95.4%	1.3%
**Ir-3**	HOMO	40.0%	47.2%	12.8%
	LUMO	2.7%	94.7%	2.6%

Table S5. The reported device performances with Ir(III) complexes based on sulfur-contained four-membered ancillary ligands.

**Structure**	**CIE (x,y)**	L_{max} **[cd m^{-2} (V)]**	$\eta_{c,max}$ **(cd/A)**	$\eta_{p,max}$ **(lm/W)**	**EQE$_{max}$ (%)**	**Reference**
	–	2525 (13)	2.9	1.8	–	*Synth. Met.*, **2005**, *152*, 225
	–	635 (17.5)	1.08	2.40	–	*J. Organomet. Chem.*, **2006**, *691*, 3519

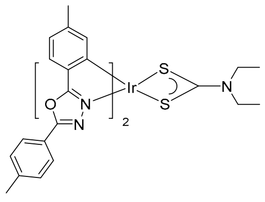	–	339 (13.8)	1.24	1.36	–	*J. Organomet. Chem.*, **2006**, *691*, 3519
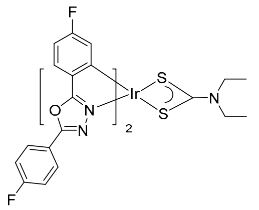	(0.16, 0.27)	22250 (22.5)	9.88	2.38	–	*Chem. Commun.*, **2007**, 1352
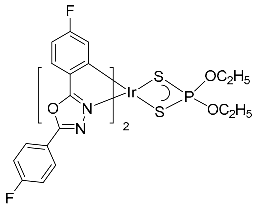	(0.14, 0.25)	3659 (21.5)	5.41	1.01	–	*Chem. Commun.*, **2007**, 1352
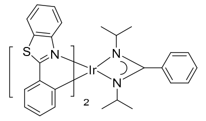	(0.64, 0.36)	30160 (9)	18.1	18.4	15.4	*J. Mater. Chem.*, **2009**, *19*, 8072
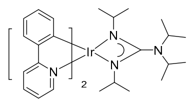	(0.48, 0.51)	110 400 (9.5)	–	93.6	25.3	*J. Mater. Chem. C*, **2013**, *1*, 2920
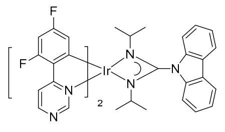	(0.27, 0.58)	100950 (8.5)	–	68.8	17.6	*Adv. Funct. Mater.*, **2014**, *24*, 7420
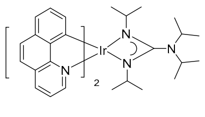	(0.57, 0.41)	91030 (11)	–	61.5	22.5	*Adv. Funct. Mater.*, **2014**, *24*, 7420
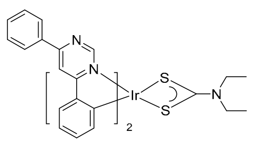	(0.4745 , 0.5109)	81918	30.12	–	9.28	*RSC Adv.*, **2016**, *6*, 64003
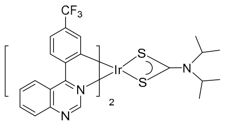	(0.68, 0.31)	33540	11.89	8.87	19.46	*Chem. Sci.*, **2019**, *10*, 3535
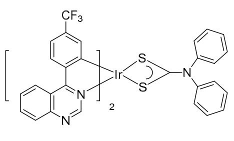	(0.65, 0.33)	46920	28.95	20.97	28.10	*Chem. Sci.*, **2019**, *10*, 3535

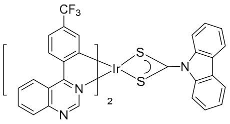	(0.63, 0.36)	60950	40.68	33.63	30.54	*Chem. Sci.*, **2019**, *10*, 3535
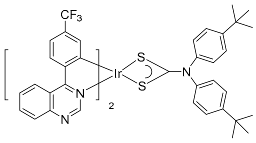	(0.662, 0.330)	44080	21.75	17.45	24.38	*J. Mater. Chem. C*, **2019**, *7*, 3862
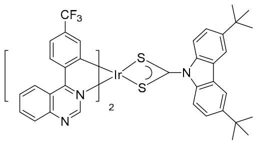	(0.634, 0.363)	65240	34.72	27.97	26.66	*J. Mater. Chem. C*, **2019**, *7*, 3862
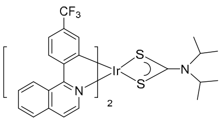	(0.657, 0.339)	34200	18.67	20.69	18.11	*Mater. Chem. Front.*, **2019**, *3*, 860
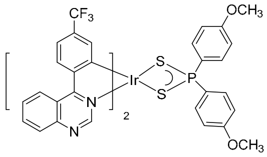	(0.65, 0.33)	25687	24.83	18.13	19.20	*Dalton Trans.*, **2019**, *48*, 7583
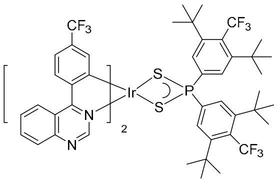	(0.65, 0.34)	26184	30.24	22.61	21.50	*Dalton Trans.*, **2019**, *48*, 7583
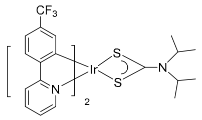	(0.253, 0.634)	33466	86.81	59.85	25.71	*J. Mater. Chem. C*, **2019**, *7*, 7273
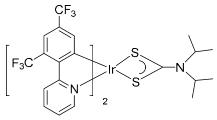	(0.346, 0.615)	38403	66.91	40.23	17.30	*J. Mater. Chem. C*, **2019**, *7*, 7273
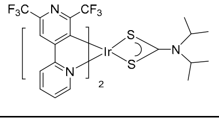	(0.191, 0.533)	38812	86.59	52.71	31.24	*J. Mater. Chem. C*, **2019**, *7*, 7273
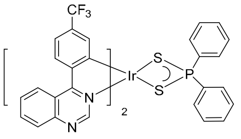	(0.65, 0.33)	27202	26.15	16.42	19.50	*New J. Chem.*, **2019**, *43*, 8722

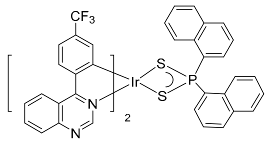	(0.64, 0.34)	43643	31.32	22.35	19.50	*New J. Chem.*, **2019**, *43*, 8722
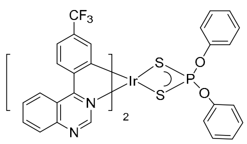	(0.61, 0.38)	32717	44.29	33.92	20.30	*J. Mater. Chem. C*, **2019**, *7*, 6972
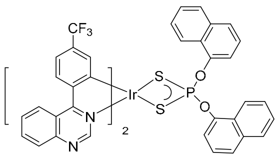	(0.59, 0.39)	52030	57.98	44.40	24.90	*J. Mater. Chem. C*, **2019**, *7*, 6972
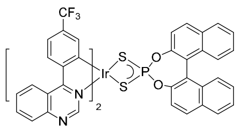	–	34 986	54.6	45.3	23.7	*Chem. Commun.*, **2019**, *55*, 8215
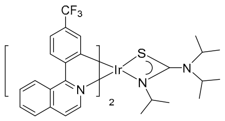	(0.66, 0.31)	4222	9.4	8.2	12.0	*J. Mater. Chem. C*, **2020**, *8*, 1391
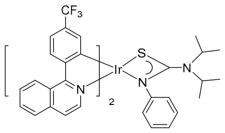	(0.68, 0.32)	6334	7.4	5.3	10.2	*J. Mater. Chem. C*, **2020**, *8*, 1391
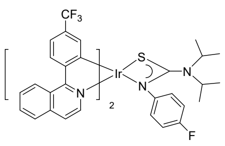	(0.67, 0.32)	6982	10.5	8.4	13.1	*J. Mater. Chem. C*, **2020**, *8*, 1391
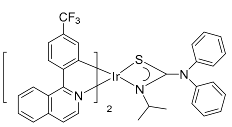	(0.66, 0.32)	3534	10.0	8.2	11.6	*J. Mater. Chem. C*, **2020**, *8*, 1391
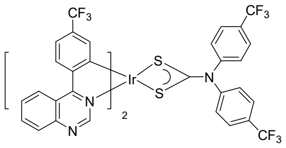	(0.61, 0.36)	30740	46.86	37.73	26.10	*J. Mater. Chem. C*, **2020**, *8*, 7411
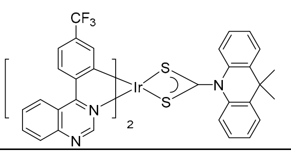	(0.62, 0.34)	14080(13.3)	10.86	4.70	8.8	This paper

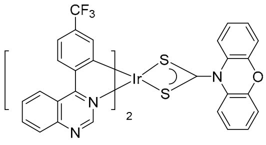	(0.64, 0.35)	18740(10.3)	15.23	8.04	11.0	This paper
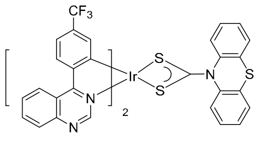	(0.63, 0.35)	22480(10.2)	23.71	16.23	18.1	This paper
